# Can a tailored implementation programme enhance the adoption of guideline-adherent behaviour in physiotherapists and chiropractors managing patients with low back pain? An implementation study

**DOI:** 10.1186/s43058-025-00820-y

**Published:** 2025-12-06

**Authors:** Maja Husted Hubeishy, Jeanette Trøstrup, Malene Joensen, Kristin Thomas, Petra Dannapfel, Camilla Blach Rossen, Tue Secher Jensen, Thomas Maribo, Nanna Rolving

**Affiliations:** 1 University Clinic for Interdisciplinary Orthopaedic Pathways (UCOP), Elective Surgery Centre, Silkeborg Regional Hospital, Silkeborg, Denmark; 2 Department of Public Health, Aarhus University, Aarhus, Denmark; 3 Department of Physiotherapy and Occupational Therapy, Aarhus University Hospital, Palle Juul‑Jensens Boulevard 99, Aarhus N, 8200 Denmark; 4 The Danish Healthcare Quality Institute (DHQI), Aarhus, Denmark; 5 Department of Health Science and Technology, Aalborg University, Aalborg, Denmark; 6 Department of Health, Medicine and Caring Sciences, Linköping University, Linköping, Sweden; 7 Department of Clinical Medicine, Aarhus University, Aarhus, Denmark; 8 Medical Diagnostic Centre, University Clinic for Innovative Patient Pathways, Regional Hospital Central Jutland, Silkeborg, Denmark; 9 Radiology Department, Regional Hospital Central Jutland, Silkeborg, Denmark; 10 Department of Sports Science and Clinical Biomechanics, University of Southern Denmark, Odense, Denmark; 11 DEFACTUM, Central Region Denmark, Aarhus, Denmark

**Keywords:** Implementation programme, Physiotherapists, Chiropractors, Low back pain, Biopsychosocial approach, Guidelines, Evidence-based practice

## Abstract

**Background:**

Low back pain (LBP) is the primary contributor to disability worldwide, leading to a significant healthcare burden. Implementing evidence-based practice (EBP) can reduce this burden, as healthcare professionals (HCPs) working evidence-based reduce the number of treatments, the use of imaging and medication compared to HCPs not working evidence-based. Clinical practice guidelines have been published to help HCPs implement research evidence into practice. Unfortunately, studies have consistently shown a lack of adherence to guidelines in LBP care. This study aimed to investigate the implementation outcomes of a tailored programme for implementing key recommendations from LBP guidelines among Danish physiotherapists and chiropractors working in primary care.

**Methods:**

This study was conducted as a 'pre-post' implementation study among 80 physiotherapists and chiropractors from 15 primary care clinics. The implementation object was the two key guideline recommendations: 1) screening of psychosocial risk factors and 2) patient education, including reassuring information. The programme comprised multipronged strategies and was designed as a step-by-step implementation process comprising 16 hours of activities distributed over 16 weeks. Adoption was measured as changes in self-reported behaviour and perceptions of the professional role and culture from baseline to follow-up at 16 weeks. Acceptability and appropriateness of the programme were measured weekly using a four-point Likert scale. Feasibility was measured at 16-week follow-up using a five-point Likert scale. Fidelity was measured as the number of strategies and implementation support delivered as planned and registered by the research team.

**Results:**

An increase in the adoption of screening of psychosocial factors and offering patient education, including reassuring information, was seen in 38% and 33% of participants, respectively. Most participants reported that the programme was partly or overall acceptable and appropriate. The feasibility of the implementation programme was assessed as moderate to high, and the fidelity of the implementation programme was determined as high.

**Conclusion:**

The tailored implementation programme enhanced the adoption of the guidelines and changed the participants’ professional identity and culture. Most participants found the programme partly or overall acceptable and appropriate. The programme was feasible, but the perspective requires refinements, as most participants rated it moderately feasible.

**Trial registration:**

Central Denmark Region, Registered November 11, 2021, act no. 1–16-02–93-19.

**Supplementary Information:**

The online version contains supplementary material available at 10.1186/s43058-025-00820-y.

Contributions to the literature

- Research highlights a significant gap in the implementation of clinical practice guidelines for the management of low back pain.
- This study demonstrates that a tailored multipronged implementation programme (webinars, e-learning videos, communication exercises, peer learning and group dialogue meetings) increased guideline adoption.
- The programme enhanced physiotherapists' and chiropractors' self-reported behaviours, professional identity, and culture by increasing their skills and confidence in using a biopsychosocial approach in assessing and treating patients with low-back pain.
- The programme's iterative approach, emphasising small step-by-step changes, fostered self-reflection, promoting the clinicians' self-awareness of their practices, and strengthened social relatedness within clinics.

## Background

Low back pain (LBP) is the primary contributor to disability worldwide, leading to a significant healthcare burden [1]. One way to reduce the burden of disease is by implementing evidence-based practice (EBP), as the literature shows that healthcare professionals (HCPs) working evidence-based reduce the number of treatments, the use of imaging and the use of medication compared to HCPs not working evidence-based [2–5]. Providing EBP requires that HCPs implement a combination of three elements into their practice: 1) research evidence (evidence from clinical research), 2) the patient's preference and behaviour, and 3) the patient's circumstances. Once these components are known, HCPs can combine them with their clinical expertise and make an evidence-based decision, i.e. apply EBP [6]. In most countries, health authorities have developed and published clinical guidelines to help HCPs implement research evidence into practice [7]. The clinical practice guidelines provide the latest research evidence within a defined area to help bridge the gap between practice and research. Unfortunately, studies have consistently shown a lack of implementation of guidelines within LBP management [8–11].

Providing EBP for patients with LBP has required a paradigmatic shift in the HCPs' approach from a biomedical to a biopsychosocial focus [12–14]. With undergraduate training typically having a biomedical focus, HCPs are predominantly educated and trained to identify the source of pain, that could explain the patient's pain [15, 16]. However, solid research evidence has established that the occurrence and progression of LBP is influenced not only by biomedical factors but also by psychological (maladaptive pain beliefs, depression, and anxiety) and social (financial, family, and work-related issues) factors [17, 18]. Studies have shown that applying a biopsychosocial approach in LBP management decreases patients' disability and increases their quality of life [19, 20]. Despite this being known for decades, HCPs are still challenged in adopting a biopsychosocial approach and changing their professional behaviour according to this [16, 21]. The lack of implementation of a biopsychosocial approach within LBP management may be based on deeply rooted barriers, requiring changes to the identity and culture of the HCPs [22, 23]. Although implementing a biopsychosocial approach is complex, some studies have succeeded by using extensive implementation strategies [24, 25]. Nevertheless, there is conflicting evidence about the ideal implementation strategies, as other studies have shown no success [26–28]. What is known from implementation science is that tailoring the strategies to the context is critical to the implementation process [29, 30]. Tailoring the implementation strategies entails involving stakeholders working where the desired behaviour change is planned, as it enables the identification of barriers and facilitators to the desired behaviour. Subsequently, the barriers and facilitators must be linked to effective strategies based on behaviour change theory [31–33]. Finally, implementation science has also shown that the duration of the programme is crucial for the behaviour change to be successful [34]. Studies demonstrate significantly stronger effects on sustained behaviour change when participants are given time to practice the desired behaviour, compared to intensive strategies implemented over a few days [34].

In Denmark, previous studies have shown poor adoption of clinical practice guidelines for LBP and neck pain among physiotherapists and chiropractors [8, 35]. Therefore, an implementation project was initiated to enhance the adoption of clinical practice guidelines, entailing three phases. In phase one, a qualitative study explored the contextual barriers to implementing the LBP guidelines [36]. The study found two key barriers: 1) scepticism due to doubts about the validity and applicability of the guidelines, and 2) a deeply rooted biomechanical professional identity conflicting with the guideline recommendations about taking a biopsychosocial approach [36]. In phase two, a tailored, theory-driven implementation programme was developed in collaboration with physiotherapists and chiropractors, addressing the identified barriers using the Behaviour Change Wheel and COM-B (Capability, Opportunity, Motivation- Behaviour) [37, 38]. The implementation programme was designed to enhance the adoption of the two key guideline recommendations: 1) screening of psychosocial risk factors and 2) patient education, including reassuring information (henceforth referred to as target behaviours). The programme comprised five strategies (webinars, e-learning videos, communication exercises, peer learning, and group dialogue meetings) and additional implementation support [37]. This paper reports on the third phase of the implementation project, aiming to investigate the implementation outcomes of the tailored, theory-driven, multipronged programme for implementing key recommendations from the LBP guidelines among Danish physiotherapists and chiropractors working in primary care using a pre-post implementation study design.

Specifically, the objectives were:

1. To measure the physiotherapists' and chiropractors' *adoption* of the following target behaviours from baseline to 16-week follow-up: a) screening of psychosocial risk factors and b) offering patient education, including reassuring information.
2. To measure the physiotherapists' and chiropractors' *adoption* of a) a biopsychosocial professional identity and b) a biopsychosocial culture from baseline to 16-week follow-up.
3. To determine the physiotherapists' and chiropractors' perceptions of the programme's strategies regarding *acceptability*, *appropriateness*, and *feasibility* during implementation.
4. To determine the *fidelity* of the implementation programme as delivered by the research team.

## Methods

### Design

This study was a pre-post implementation study evaluating a tailored, theory-driven, multipronged implementation programme. The implementation programme was developed based on co-design and theory-driven processes [39]. The reporting of this study follows the STaRI guideline (see Additional file 1) [40].

### Context

The targeted sites for the implementation programme were a representative sample of 107 physiotherapy clinics and 59 chiropractic clinics in the Central Denmark Region. The Central Denmark Region comprises 19 municipalities, where 1.3 million citizens reside. In Denmark, healthcare services are tax-financed, and most are free. However, public health insurance covers only 40% and 20% of the costs in private physiotherapy and chiropractic clinics, respectively. Patients do not have direct access to physiotherapy consultations in primary care. However, they must be referred by their general practitioner or the secondary sector (hospital) to be reimbursed. In contrast, patients consulting chiropractors have direct access to their services without prior referral.

### Study population

Physiotherapy and chiropractic clinics were eligible to participate in the study if 1) at least two physiotherapists or chiropractors from the clinic agreed to participate in the 16-week intervention, and if they managed at least one patient with LBP per week; 2) a champion was selected at the clinic, who attended in a mandatory preparatory meeting and agreed to act as a facilitator throughout the intervention. Physiotherapists and chiropractors were invited to participate through an invitation video shared on LinkedIn and Facebook social media platforms, with the first author's (MHH) contact information provided. The video was developed by the first (MHH), second (JT), third (MJ) and last author (NR). Also, an invitation was sent by email from the practice consultants for physiotherapy and chiropractic (employed by the region to support quality development in primary care) to all physiotherapists and chiropractors working in primary care in the Central Denmark Region (an estimated 900 physiotherapists and 150 chiropractors in total, located at 107 physiotherapy clinics and 59 chiropractic clinics). Interested clinics were offered a telephone call or a visit, during which they received further information about the project. The clinics could register for the project from November 2022 to February 2023. None of the participants received any financial incentives or compensation for their participation, except the champions, who received a fee of 2.000 DKK for participating in the preparation meeting and for spending time printing and hanging up reminders, etc., over the 16 weeks.

### The implementation object

The implementation object of this study was the two target behaviours selected from the Danish clinical practice guideline "Treatment of new-onset low back pain", developed in 2019 [41]. See the guideline description of the two selected target behaviours (recommendations) in Table 1.

**Table 1:** Description of the two target behaviours from the Danish low back pain guideline [41]

Description of target behaviour 1: Screening of psychosocial risk factors
• Consider including questions regarding psychological and social issues in the medical history, as these provide an opportunity to identify patients with problems that require special attention
Description of target behaviour 2: Patient education, including reassuring information
• Consider offering individualised patient education for patients with new-onset low back pain in addition to usual treatment, where this is considered to increase self-care
• Consider offering individualised patient education to patients who are concerned or anxious about their low back pain or who become inactive or behave passively in connection with the pain
• Patient education should include a reassuring dialogue that contains elements of cognitive and behavioural techniques and should be individualised to change the individual patient´s inappropriate thoughts, feelings, and behaviour

**Fig. 1:**
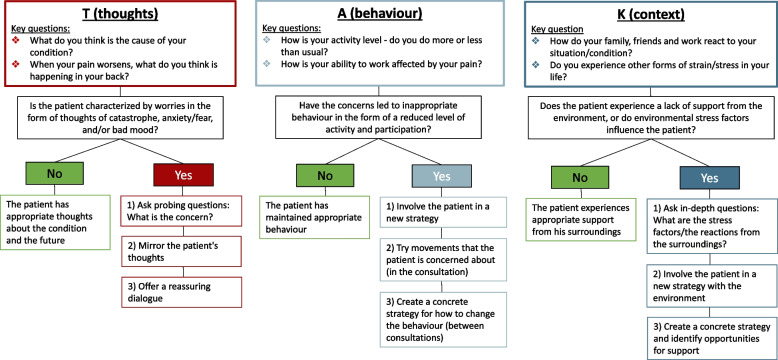
The flowchart of the TAK model specifying the target behaviours

The target behaviours were specified using the communication model shown in Fig. 1: The so-called "TAK model – thank you for asking, with the Danish word for thank you being TAK "Tanker (thoughts) Adfærd (behaviour) Kontekst (context) [37]. The TAK model was developed during Phase two by MHH and experts in the field to guide physiotherapists and chiropractors (hereafter referred to as participants) through screening patient's psychosocial risk factors using suggested questions. Further, the TAK model offered guidance on how to offer patient education, including reassuring information, for patients with maladaptive thoughts and/or behaviours and/or context. Details about the specification of the target behaviours are shown in the TAK model flowchart in Fig. 1 and Additional files 2–4. Briefly described the steps are as follows: In the first step of the TAK model, the participants were guided to screen the patients' thoughts (T = Thoughts) by asking questions regarding what the patients believed was the cause of the pain and what came to their mind when the pain increased. If a patient had maladaptive thoughts about their LBP, the participants were trained to offer patient education by mirroring their maladaptive thoughts and offering a reassuring dialogue. Subsequently, the participants were educated and trained to screen whether the patients' maladaptive thoughts led to undesirable behaviours (A = behaviour) by asking whether the patient did more or fewer activities than usual and how he/she handled the pain. If a patient's behaviour was unhelpful, the participants could encourage patients to try behaviours (or movements) they were concerned about during the consultation while participants gave reassuring information. Lastly, the participants were educated and trained to ask whether the patients experienced a lack of support or environmental stress from their context (K = context), e.g., illness in the family. Participants were educated and trained to involve patients negatively affected by their context in creating new strategies to manage the environment better. It was specified that the two target behaviours should be applied to all patients with LBP during the first consultation and continue throughout the treatment course.

### The implementation programme

The implementation programme was developed to enhance the adoption of the target behaviours. The development of the implementation programme (phase 2) has been described in detail elsewhere [37]. In brief, the programme was developed using the Behaviour Change Wheel [38], which emphasises the need to involve participants in identifying local barriers and linking intervention functions (broad categories of means by which an intervention/strategy can change behaviour) and behaviour change techniques to these barriers. The target behaviours were designed to be implemented in a step-by-step programme with 16 hours of education and training spread over 16 weeks (See Table 2). The delivery of the programme was determined based on the participants' recommendation to implement the TAK model in small steps, as they had experienced too many ineffective weekend courses. The use of one hour per week would be manageable for most, and a presentation of "only" having to use the equivalent of a two-day course (16 hours) spread over four months was therefore deemed a relevant implementation. The implementation programme consisted of five strategies: webinars, e-learning videos, communication exercises, peer learning, and group dialogue meetings, which were used repeatedly over the 16 weeks. The participants could perform all the strategies at their clinics. During the first two weeks, the programme focused on introducing the target behaviours (explaining why, what, and how to screen and how to offer patient education) and on having participants reflect on their current behaviours. The reflection included how the participants communicated with patients about treatment expectations and their clinician role. Reflections were facilitated through participation in a webinar, an e-learning video, and a communication exercise. Following the two introductory weeks, specific training in applying the TAK model began, with three weeks of use for each part of the model (Thoughts, Behaviour, Context). The first week in each part was spent watching an e-learning video (demonstrating why and how to perform the target behaviours). After watching the video, participants had to complete a communication exercise to train how to practice the target behaviours shown in the video while adapting the communication to their practice. During the second week in each part, the participants performed a peer learning exercise, alternately giving/receiving feedback to/from a colleague concerning adopting the target behaviours. Each clinic held a group dialogue meeting in the last week of each part, where successes and challenges in implementing the target behaviours were discussed. Thus, the four strategies, e-learning videos, communication exercises, peer learning, and group dialogue meetings were held in all three parts of the model. The programme also consisted of implementation support that facilitated the behaviour change and consisted of a champion from every clinic, a physical material folder containing weekly descriptions of the activities, a weekly email reminder of the coming week's activities, a specially designed website holding all intervention material [42], and a visit from an implementation consultant once during the implementation period.

**Table 2:** The content and construction of the 16-week implementation programme

Week	Strategy	Objective/Mechanism	TAK domain	Expected time consumption
0	Champion introduction meeting	Prepare support by informing about content and activities for the coming 16 weeks.	All three domains	1.5 h (on-site)
1	Webinar 1	Increase knowledge about the target behaviours through education by providing information about the evidence, why the behaviours are essential.	All three domains	1.5 h (online or on the webpage when it fits you)
2	E-learning video 1 + Communication exercise 1	Reflection and communication exercise about one’s roles and patient expectations.	All three domains	1.0 h (with one or more colleagues)
3	E-learning video 2	Providing a demonstration of how to perform the behaviour regarding the patients’ Thoughts.	Patients Thoughts (Tanker)	1.0 h (with one or more colleagues)
	Communication exercise 2	Training communication skills in a safe environment with colleagues, where performance is not yet necessary, to discuss patients' Thoughts.		
4	Peer learning 1	Giving/receiving feedback on behaviour to promote reflection on one's role and behaviour in connection with screening, and provide patient education regarding the patients' Thoughts.		1.0 h (with one or more colleagues)
5	Group Dialogue Meeting 1	Improving relatedness and facilitating acceptance of new behaviour/culture of change. Also, sharing successes and challenges in conducting screening and patient education regarding patients' Thoughts, as well as collaborating on the terminology used in patient records.		1.0 h (with all participants from the clinic)
6	Visits from an implementation consultant	Support from an implementation consultant, who will provide tailored support to meet the participant's specific needs.	Patients Thoughts	30 min.—1.0 h (voluntarily – the consultant is visiting the clinic for 2 h)
7	Easter holiday			
8	Question hour	Offering support and allowing participants to ask questions about the target behaviours.	Patients Thoughts	30 min (voluntary online)
9	E-learning video 3 + 3B	Providing a demonstration of how to perform the behaviour regarding the patients’ Behaviour.	Patients Behaviour (Adfærd)	1.0 h (with one or more colleagues)
	Communication exercise 3	Training communication skills in a safe environment with colleagues, where performance is not yet necessary, to discuss patients Behaviour.		
10	Peer learning 2	Giving/receiving feedback on behaviour to promote reflection on one's role and behaviour in connection with screening, and provide patient education regarding the patients' Behaviour.		1.0 h (with one or more colleagues)
11	Group Dialogue Meeting 2	Improving relatedness and facilitating acceptance of new behaviour/culture of change. Also, sharing successes and challenges in conducting screening and patient education regarding patients' Behaviour, as well as collaborating on the terminology used in patient records.		1.0 h (with all participants from the clinic)
12	Webinar 2	Increase knowledge about the target behaviours: screening and offering patients education regarding the patients’ Context by providing information about the evidence and why the behaviour is essential.	Patients Context (Kontekst)	hour (on the webpage, see it when it fits you)
13	E-learning video 4 + 4B	Providing a demonstration of how to perform the behaviour regarding the patients’ Context.		1.0 h (with one or more colleagues)
	Communication exercise 4	Training communication skills in a safe environment with colleagues, where performance is not yet necessary, to discuss patients Context.		
14	Peer learning 3	Giving/receiving feedback on behaviour to promote reflection on one's role and behaviour in connection with screening, and provide patient education regarding the patients' Context.		1.0 h (with one or more colleagues)
15	Group Dialogue Meeting 3	Improving relatedness and facilitating acceptance of new behaviour/culture of change. Also, sharing successes and challenges in conducting screening and patient education regarding patients' Context, as well as collaborating on the terminology used in patient records.		1.0 h (with all participants from the clinic)
16	Future Plan Meeting	Supporting the sustainability of the target behaviours involved facilitating the participants' decision on which strategies to maintain after the intervention, how they should be implemented, and who was responsible.	All three domains	1.0 h (with all participants from the clinic)

### Data collection

The data were collected via electronic questionnaires sent by email or text to all participants. The questionnaires were designed specifically for the current study using Research Electronic Data Capture (REDCap) hosted at Aarhus University [43]. They were developed and pilot-tested in January 2023 in collaboration with researchers and HCPs at Aarhus University Hospital and primary care physiotherapists. Questionnaires included items measuring target behaviours, professional identity (e.g., perceptions of their role as a clinician and feelings of skill), and the culture at the clinic. Questionnaires were emailed to participants at baseline and at the 16-week follow-up (see Table 3). The participants also answered short questionnaires regarding the acceptability, appropriateness, and feasibility of the various implementation strategies sent weekly by text message over the 16 weeks; see an example in Additional file 5.

**Table 3:** Elements defining the target behaviours, biopsychosocial professional identity and culture

**Target behaviours**	**Response items for all elements**
Do you *screen* your patients' psychological and social issues?	◦ do not know ◦ not at all ◦ to a low extent ◦ to a moderate extent ◦ to a high extent
Do you *guide* your patients in managing their psychological and social issues?	
**Biopsychosocial professional identity**	
Do you believe it is your *role* to involve your patients' psychological and social issues?	
Do you have the sufficient *skills* to screen your patients' psychological and social issues?	
Do you have the sufficient *skills* to guide your patients in managing psychological and social issues?	
Do you *feel confident* in involving your patients' psychological and social issues?	
Are you *interested* in involving your patients' psychological and social issues?	
**Biopsychosocial culture**	
Do you experience your *colleagues involving* their patients' psychological and social issues?	
Do you engage in or request *professional back-and-forth* with your colleagues about your patients' psychological and social issues?	

### Outcomes

#### Adoption of target behaviours (objective 1)

The adoption of the target behaviours was measured by asking the participants at baseline and again at the 16-week follow-up to what extent they believed they a) screened their patients’ psychosocial risk factors and b) offered patient education (see Table 3). The target behaviours were measured on a five-point Likert scale: 1) do not know, 2) not at all, 3) to a low extent, 4) to a moderate extent, and 5) to a high extent. However, the 'do not know' answers were removed from the adoption analysis.

*Pre-adopters* were defined as participants who reported a ‘high extent’ of the behaviour at baseline. *Pre-adopters* were excluded from the adoption analyses, as they were already performing the target behaviour.

*Adopters* were defined as participants who reported to perform the target behaviours to a greater extent at 16-week follow-up than at baseline, such as altering from 'moderate' to a 'high extent' in screening patients' psychosocial risk factors or offering patient education.

*Non-adopters* were defined as participants who reported no change (at levels below high extent) or a negative change, such as altering from 'moderate' to 'low extent' from baseline to follow-up.

The definition of adoption at follow-up is shown in Table 4, with dark grey areas illustrating adopters, white areas non-adopters and light grey areas pre-adopters.

**Table 4:**
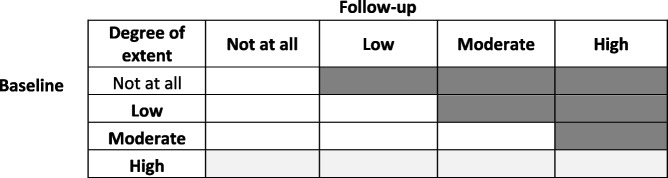
Illustrating the dark grey areas as the definition of adoption at follow-up

The dark grey areas represent adopters, and the white areas represent non-adopters. The light grey areas represent pre-adopters, who were excluded from the adoption analysis

#### Adoption of identity and culture (objective 2)

Participants reporting 'high extent' at baseline were defined as *Pre-adopters* and excluded from the adoption analyses.

*Adoption *of a) biopsychosocial professional identity and b) biopsychosocial culture was defined as a positive change from baseline to 16-week follow-up (e.g. moving from 'moderate' to a 'high extent' in feeling it as their role). Participants who reported no change (at levels below high extent) or a negative change, such as altering from 'moderate' to 'low extent' from baseline to follow-up, were defined as *Non-adopters*. The biopsychosocial professional identity was determined based on five elements, and the biopsychosocial culture was based on two elements (see Table 3). The professional identity and culture were measured using the same five-point Likert scale to measure the target behaviours.

#### Acceptability, appropriateness, and feasibility (objective 3)

*Acceptability* was defined as the extent to which participants found the content and format of the four strategies: webinars, e-learning videos, peer learning, and group dialogue meetings to be acceptable. *Appropriateness* was defined as the extent to which participants found the four strategies: webinar, communication exercise, peer learning, and group dialogue meeting, to be appropriate for their daily practice. Acceptability and appropriateness were measured weekly during the implementation programme using a four-point Likert scale of 1) yes, overall acceptable/appropriate; 2) yes, partly; acceptable/appropriate; 3) not acceptable/appropriate; 4) do not know.

*Feasibility* was defined as to what extent the implementation programme was feasible for the participants to complete based on three parameters:

1. Compliance: the number of participants participating in the five strategies, measured as self-reported participation in weekly text messages (yes, no).
2. Participants' assessment of the implementation programme's feasibility as a whole.
3. Participants' assessment of the implementation support (champions, webpage, material folder, status emails, visit) contributes to accomplishing the programme.

The participants' assessment (parameters 2 and 3) was measured as a self-reported outcome at the 16-week follow-up using the same five-point Likert scale used to measure the target behaviours in objective 1.

#### Fidelity (objective 4)

Fidelity was defined as the extent to which the research team delivered the implementation programme (all five strategies and implementation support) as planned. Fidelity was measured as the number of strategies and implementation support delivered as planned and registered by the research team.

### Analysis

Data were analysed using STATA 18 (Stata Corp, College Station, TX). Descriptive statistics (mean [SD] for continuous variables and frequencies [percentage] for categorical variables) were used to describe the participants' characteristics.

The adoption of the target behaviours (objective 1) and the biopsychosocial professional identity and culture (objective 2) was analysed by measuring the proportion of non-adopters at baseline who adopted the behaviour at follow-up (reported a positive change in behaviour).

The acceptability, appropriateness, feasibility (objective 3) and fidelity (objective 4) were analysed using descriptive statistics for categorical variables.

## Results

### Participants

Twenty-eight clinics responded to the invitation, of which two clinics were excluded due to not being primary care clinics, three clinics responded after the deadline, and eight clinics decided not to participate after having received an explanation of the content and structure of the course by e-mail, telephone, or visits, resulting in 15 participating primary care clinics, comprising 74 physiotherapists and six chiropractors.

Table 5 shows the characteristics of the participants and clinics. Overall, there was an even gender distribution (53% males). Most were physiotherapists (93%) and self-employed (49%). They had an average of 14,1 years of clinical experience, and approximately half (48%) had undergone further training in communication within pain management. The clinics were equally distributed between rural and urban areas. Most clinics (60%) were medium-sized, comprising 5–9 HCPs.

**Table 5:** Descriptive characteristics of participants and clinics

Variables	Participants (*n*=80)
Sex, n (%)	
Male	42 (53)
Professions, n (%)	
Physiotherapists	74 (93)
Age, mean (range)	43 (26–70)
Years of clinical experience, mean (range)	
Physiotherapists	14,2 (1–42)
Chiropractors	14,0 (7–32)
Years working at the present clinic, mean (range)	10 (0–32)
Work role at the clinic, n (%)	
Owner	14 (18)
Manager	3 (4)
Self-employed (renting at clinic)	39 (49)
Employed	28 (35)
Other	1 (1)
Number of treated patients with LBP per week, n (%)	
1–5 patients	43 (54)
6–10 patients	23 (29)
11 or more patients	14 (18)
Number of participants with further education/training within communication and pain management, n (%)	38 (48)
Variables	Clinics (*n*=15)
Geographical location*, n (%)	
Rural	7 (47)
Urban	8 (53)
Size of clinic**, n (%)	
Small	2 (13)
Medium	9 (60)
Large	4 (27)

*Rural area is defined as a population of less than 10,000, and Urban area as a population of 10,000 or more

**A small clinic comprises 1–4 healthcare professionals (HCPs), a medium clinic comprises 5–9 HCPs, and a large clinic comprises 10 or more HCPs

### Adoption of target behaviours (objective 1)

A group of participants, 15% and 14%, respectively, reported ‘a high extent’ of the target behaviours from baseline and were therefore defined as pre-adopters. Of the remaining participants (non-adopters at baseline), 38% and 33% adopted the two target behaviours, screening for psychosocial risk factors and patient education, respectively, as shown in Table 6 and elaborated in Additional file 6. However, 57% and 60% did not adopt the target behaviour during the 16-week implementation period, respectively.

**Table 6:** Adoption of target behaviours, biopsychosocial professional identity and culture

	Numbers	Baseline		Follow-up Only including Non-adopters from baseline		
Questions*	*n* = 80 n (%)	Pre-adopters** n (%)	Non-adopters n (%)	Adopters*** n (%)	Non-adopters n (%)	Missing
Target behaviour						
Do you screen your patients' psychological and social issues?	80 (100)	12 (15)	68 (85)	26 (38)	39 (57)	3 (4)
Do you guide your patients in managing their psychological and social issues?	66 (83)	9 (14)	57 (86)	19 (33)	34 (60)	4 (7)
Biopsychosocial identity						
Do you believe it is your *role* to involve your patients' psychological and social issues?	80 (100)	40 (50)	40 (50)	20 (50)	19 (48)	1 (3)
Do you have the sufficient *skills* to screen your patients' psychological and social issues?	80 (100)	4 (5)	76 (95)	41 (54)	32 (42)	3 (4)
Do you have the sufficient *skills* to guide your patients in managing psychological and social issues?	44 (55)	4 (9)	40 (90)	17 (43)	17 (43)	6 (15)
Do you *feel confident* in involving your patients' psychological and social issues?	79 (99)	13 (16)	66 (84)	35 (53)	31 (47)	0 (0)
Are you *interested* in involving your patients' psychological and social issues?	80 (100)	49 (61)	31 (39)	15 (48)	14 (45)	2 (6)
Biopsychosocial culture						
Do you experience your *colleagues involving* their patients' psychological and social issues?	65 (65)	8 (12)	57 (88)	17 (30)	34 (60)	6 (11)
Do you engage in or request *professional back-and-forth* with your colleagues about your patients' psychological and social issues?	80 (100)	6 (8)	74 (93)	26 (35)	46 (62)	2 (3)

^*^The participants answered the questions using a 5-point Likert scale: do not know, not at all, low extent, moderate extent or high extent

^**^Pre-adopters were excluded from the analysis because they were already reporting adopting the target behaviour at baseline

^***^Adopters were defined as participants who did not report to perform the behaviour to a high extent at baseline and who increased their use of the target behaviours, biopsychosocial identity or culture to 16-week follow-up (e.g., from 'moderate' to 'high' extent)

### Adoption of identity and culture (objective 2)

As shown in Table 6, 5–61% of the participants reported having ‘a high extent’ of biopsychosocial identity at baseline and were therefore defined as pre-adopters. Of the non-adopters at baseline, 43–54% reported adopting a biopsychosocial professional identity at follow-up, depending on the element in question. The most significant change was in the element 'being skilled to screen patients' psychosocial issues', with 54% participants adopting this element. Concerning adopting a biopsychosocial culture at the clinic, 8–12% of the participants were defined as pre-adopters. Thirty per cent of the non-adopters reported that, at follow-up, their colleagues were now addressing patients' psychosocial issues to a greater extent in their treatments, and 35% reported adopting a professional back-and-forth discussion with their colleagues about patients' psychosocial issues.

### Acceptability, appropriateness, and feasibility (objective 3)

On average, 72% of participants reported on the acceptability and appropriateness of the five strategies, with a response rate of 92% in the assessment of the first webinar. In contrast, the third peer learning session was assessed by only 53%.

**Fig. 2:**
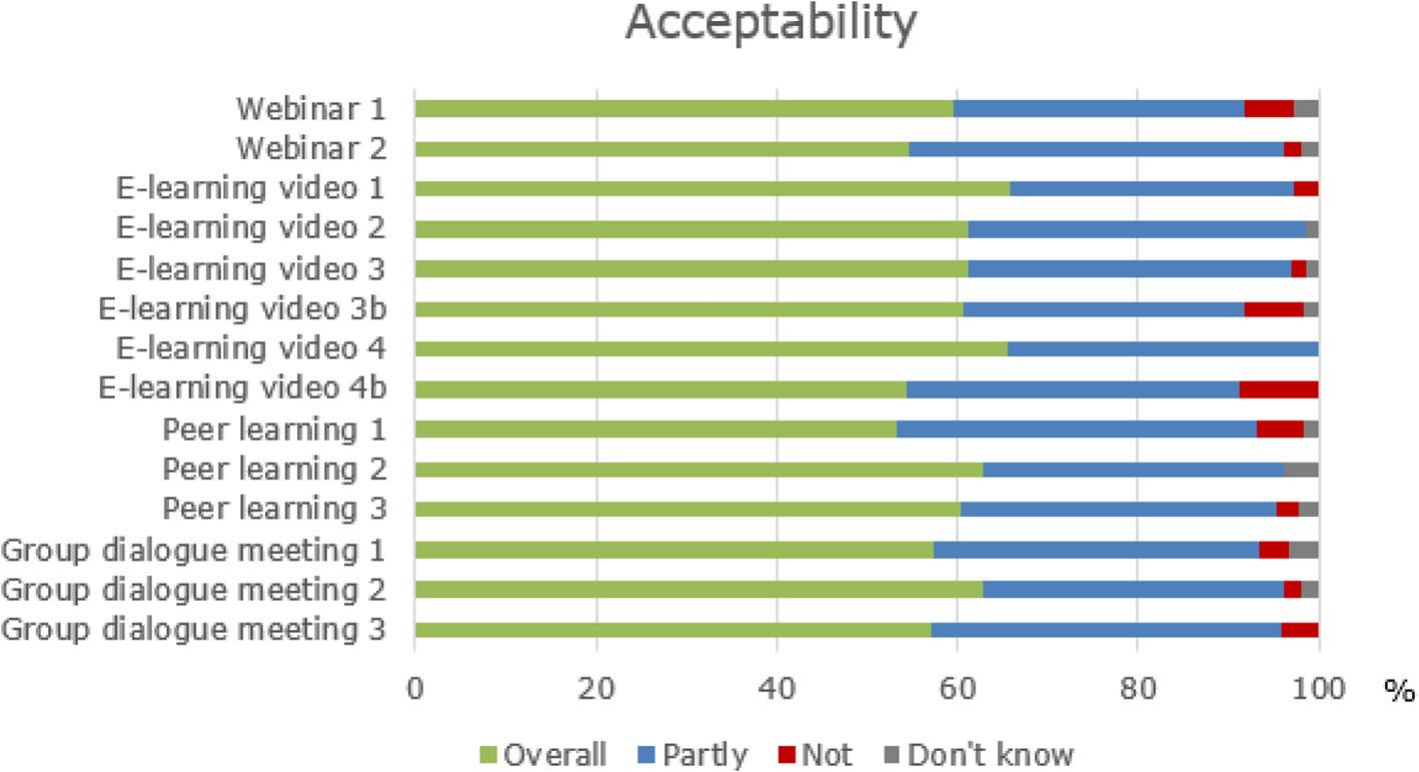
The participants' acceptability of the strategies in the implementation programme

As shown in Fig. 2, the element assessed as overall acceptable by most participants was the first e-learning video (66%). In comparison, the strategy that was reported to be overall acceptable by the least was the first peer learning session (53%). A small number of participants (0–5%) evaluated the strategies as *not* acceptable, except for e-learning videos 3B and 4B, which 7% and 9%, respectively, found *not* acceptable.

**Fig. 3:**
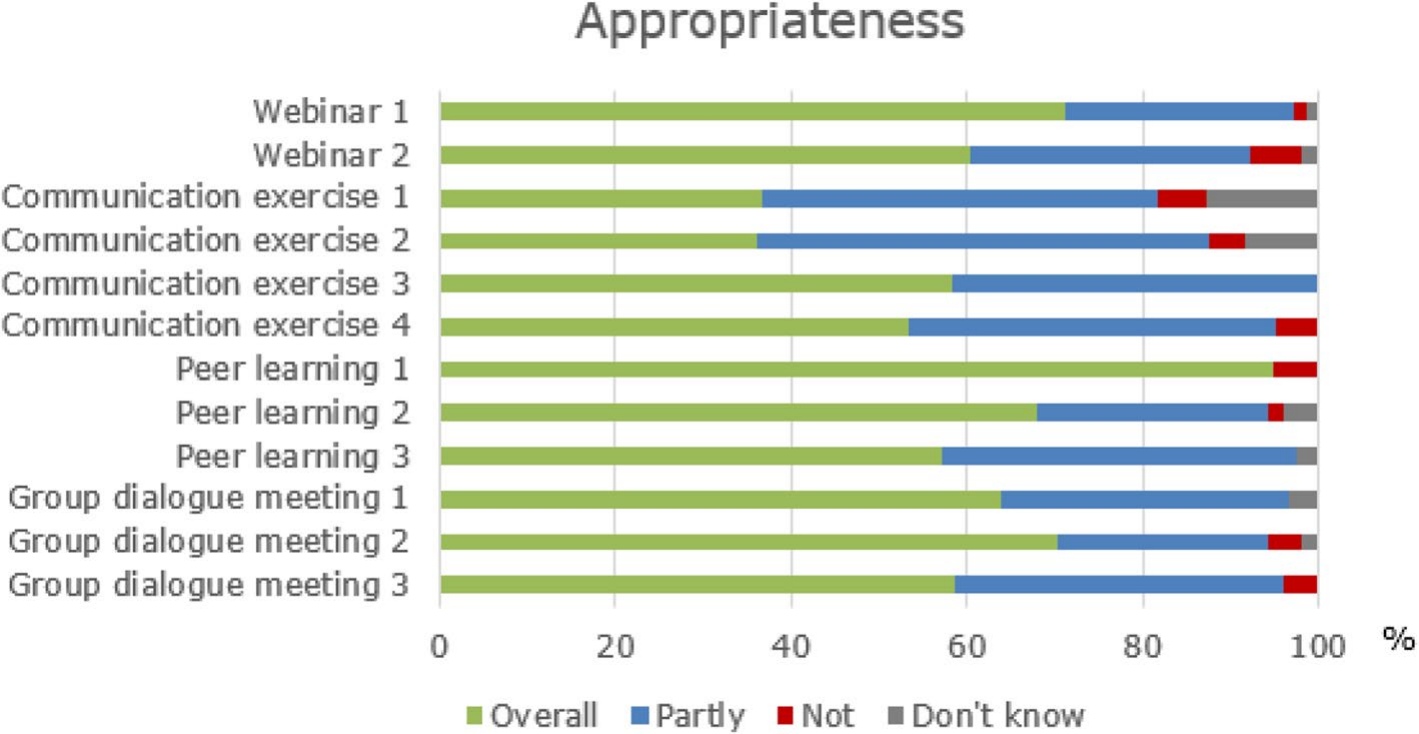
The participants' appropriateness of the strategies in the implementation programme

As shown in Fig. 3, the element assessed as overall appropriate by most participants was the first peer learning session (95%). In comparison, the strategy reported as overall appropriate by the least was the second communication exercise (36%). The four strategies were rated as *not* appropriate by a small number of the participants (0–6%).

On average, 73% of participants participated in the strategies (feasibility parameter 1). Table 7 shows that the participants' compliance with the strategies ranged from 54 to 91%, with a decreasing tendency over the 16 weeks.

**Table 7:** Compliance (number of participants participating) of the five strategies

Strategies	Participation (*n* = 80)		
	Yes	No	Missing
	n (%)		
Webinar 1 Information about why to perform all three domains (Thoughts, Behaviour, Context)	73 (91)	1 (1)	6 (8)
E-learning video 1 Information and demonstration of how to include the matching of expectations	70 (88)	4 (5)	6 (8)
Communication exercise 1 Expectation communication exercise	70 (88)	4 (5)	6 (8)
E-learning video 2 Information and demonstration on how to screen and provide patient education regarding the patients' thoughts	72 (90)	2 (3)	6 (8)
Communication exercise 2 Training communication skills for screening and patient education regarding patients' thoughts	73 (91)	1 (1)	6 (8)
Peer learning 1 Giving/receiving feedback on screening and providing patient education regarding the patients' thoughts	59 (74)	13 (16)	8 (10)
Group dialogue meeting 1 Sharing successes and challenges in screening and providing patient education regarding the patients' thoughts	60 (75)	7 (9)	13 (16)
E-learning video 3 Information on why to screen and provide patient education regarding the patients' behaviour	69 (86)	1 (1)	10 (13)
E-learning video 3b Demonstration on how to screen and provide patient education regarding the patients’ behaviour	62 (78)	8 (10)	10 (13)
Communication exercise 3 Training communication skills for screening and patient education regarding patients' behaviour	48 (60)	22 (28)	10 (13)
Peer learning 2 Giving/receiving feedback on screening and providing patient education regarding the patients' behaviour	53 (66)	16 (20)	11 (14)
Group dialogue meeting 2 Sharing successes and challenges in screening and providing patient education regarding the patients' behaviour	54 (68)	13 (16)	13 (16)
Webinar 2 Information about why and how to screen and provide patient education regarding the patients' context	53 (66)	14 (18)	13 (16)
E-learning video 4 Information on why to screen and provide patient education regarding the patients’ context	56 (70)	9 (11)	15 (19)
E-learning video 4b Demonstration on how to screen and provide patient education regarding the patients’ context	46 (58)	16 (20)	18 (23)
Communication exercise 4 Training communication skills for screening and patient education regarding the patients' context	43 (54)	21 (26)	16 (20)
Peer learning 3 Giving/receiving feedback on screening and providing patient education regarding the patients' context	43 (54)	20 (25)	17 (21)
Group dialogue meeting 3 Sharing successes and challenges in screening and providing patient education regarding the patients' context	51 (64)	11 (14)	18 (23)

Concerning the participants' assessment of the feasibility of conducting the implementation programme as a whole, 35% of participants found it highly feasible, 40% moderate, 16% minimally, 4% not feasible at all, and 5% did not know (feasibility parameter 2).

The participants' assessment of the implementation support showed that the website was found to be a highly contributing factor by most participants (55%), the champions as the second highest contributing factor (48%), the material folder by the third most (40%), status email by the fourth most (38%), and the implementation consultant visit by the fewest participants (15%) (feasibility parameter 3).

### Fidelity (objective 4)

The five strategies were delivered as planned by the research team, except for a change to the timing of webinar 2. The content of webinar 2 was intentionally decided during the implementation period so that the research team could adapt it to participants' specific knowledge needs or feedback on a given topic. It was planned to carry out webinar 2 in week 8 of the implementation period. However, this was changed to week 12, as the content was decided to focus on why and how the participants were involved in the patient's context. It was therefore connected to the last part of the TAK model concerning context. Instead, week 8 was replaced with an online question hour, during which participants could submit questions about the target behaviours or any doubts about the strategies (e.g., peer learning). A second clinic visit was cancelled to make room for webinar 2 in week 12. Thus, one out of two visits was not delivered as planned. The remaining implementation support elements were delivered as planned.

## Discussion

The overall aim of the present study was to investigate the implementation outcomes of a tailored, theory-driven programme for implementing LBP guidelines among physiotherapists and chiropractors in primary care. The study found that the implementation programme increased the adoption of two target behaviours: 1) screening for psychosocial factors and 2) patient education, including reassuring information, in 38% and 33% of participants who were non-adopters at baseline, respectively. Also, the programme enhanced the adoption of a biopsychosocial professional identity among 43–54% of participants who were non-adopters at baseline, particularly by improving participants' sense of being skilled and confident in using a biopsychosocial approach. Likewise, the programme increased the adoption of a biopsychosocial culture in 30% and 35% of clinics.

Most participants reported the strategies as acceptable and appropriate overall; however, two communication exercises were rated as only partly appropriate. The implementation programme was assessed as moderate to highly feasible, and the fidelity was determined as high as the research team delivered all five strategies as planned.

### Comparison of results and discussion of successful mechanisms

Overall, the implementation programme was designed in accordance with implementation science theories and frameworks, i.e., identification of local barriers, linking intervention functions and behaviour change techniques to these barriers, involving participants in strategy development, and using multipronged strategies over a sustained period of 16 weeks, using a step-by-step approach [37].

The results regarding the adoption of the target behaviours (38% adopting screening their patients' psychosocial risk factors and 33% adopting offering patient education) were consistent with a study by Schröder et al., which showed increased adherence (adoption) to LBP guideline recommendations [44]. The study by Schröder showed a significant effect of a multipronged implementation strategy on primary care physiotherapists' behaviour, including increased stratified care (screening for psychosocial risk factors) and patients receiving educational interventions. The multipronged strategy consisted of a two-day workshop and supporting clinical tools, such as patient brochures and chat forums, to facilitate communication between researchers and clinicians [44]. In line with the present study, Abbott et al. based the development of their strategy on the involvement of HCPs and the Behaviour Change Wheel theory [45]. Comparing the intervention functions and behaviour change techniques (i.e. the mechanisms behind the strategies) across the studies showed high similarities, with a strong emphasis on enablement, education, training, and persuasion. However, differences were seen with less emphasis on enablement and more on environmental restructuring functions in the present study compared to Abbott et al. [45]. Despite the differences in mechanisms and mode of delivery, the change in behaviour in both studies might be explained by the thorough development process leading to the removal of important locally identified barriers [29–31]. Also, the fact that both programmes were developed as multipronged can explain their improved adoption of guideline-adherent behaviour [34, 46]. A review by Herschell et al. investigating which training activities were most effective in implementing a psychosocial approach found positive results for multi-component training packages and little to no evidence for written materials or workshop interventions alone [24]. Furthermore, interactive training strategies such as role-playing, communication exercises, and peer learning, which were used in the present programme, have been shown to significantly impact learning and increase self-efficacy in managing patients with low back pain [47–50]. Also, these strategies have been widely used in other studies to broaden physiotherapists' scope of practice [50].

In line with the present results, previous studies have demonstrated increased HCPs' skills and confidence in having a biopsychosocial approach after participating in a 'psychosocial' training programme. The studies found that HCPs' ability to overcome resistance to change, a safe environment, and support from colleagues, managers, or psychologists played a crucial role in acquiring new skills [48, 51]. The positive results of the present study can thus also be explained by the fact that social and emotional support was one of the essential behavioural change techniques used in the programme. Likewise, time for reflection was necessary for acquiring new skills [48, 51].

Reflection was also found important in a critical review by Mescouto et al. [52]. The review showed that when physiotherapists were given time to reflect critically on their practice, they were better suited to and resisted their usual biomedical thinking [52]. The present study's programme format allowed participants to acquire new knowledge and skills step by step and revisit the material, giving them time for reflection. Thus, the format of the implementation programme may have contributed to the positive adoption result. A review by Mesner et al. concluded that a critical aspect of promoting behaviour change is increasing the frequency and duration of the intervention/training sessions, allowing the HCPs time for change and the opportunity to introduce the new behaviour in small steps [34]. Several other studies have also highlighted the intensity and duration of the intervention, with time between learning sessions, as being of great importance for behaviour change [16, 50, 53, 54].

To our knowledge, no other studies have addressed culture change in attempts to implement LBP guidelines. The behaviour change techniques used to increase a biopsychosocial culture align with different theories. Training the target behaviour in the interaction between the individual participant, the context in which they work, and collaboration with colleagues (incorporating the culture) optimally promotes behaviour change [50, 55]. In the present study, all five strategies were performed in the clinics (the context in which behaviour change is needed), and all strategies were carried out in collaboration with colleagues. However, the present study's short follow-up time (16 weeks) might have influenced the results (30% and 35% adopted a biopsychosocial culture) as culture change requires time. Likewise, not all physiotherapists and chiropractors at the participating clinics were part of the implementation programme, which may have negatively influenced the results, as the context, including non-participating colleagues, has a powerful influence on HCP practice. Theory shows that the clinic's culture can substantially influence HCP behaviour change more than any training programme, so it is essential to consider how to involve all HCPs at the clinics in the programme [55].

The findings of acceptability, appropriateness, and feasibility are in accordance with other studies [56, 57]. The high acceptability and appropriateness may also play an important role in the positive results, as these elements are considered leading indicators along with the feasibility of other implementation outcomes, such as adoption [58]. The flexibility (all material could be accessed on the website or in a distributed material folder anytime), convenience and time-saving (the participants could stay at the clinic and not spend time on traffic to, for example, a physical workshop) might explain the moderate to high feasibility found in the present study compared to face-to-face training [56, 59, 60]. To our knowledge, the decreasing tendency of the participants' compliance over 16 weeks (from 1% not participating in the strategies in week 1 to 26% not participating in week 14) has not been reported in other studies. However, this might be explained by the present study's innovative structure, which has a 16-hours intervention spread over 16 weeks. In contrast, other studies often deliver implementation compressed over a few days [44, 51, 61]. The declining participation may indicate that the programme is too long or that participants need a booster (motivation) to maintain the behaviour change over such a long period.

### Methodological discussion

A significant strength of this study was that the implementation programme was tailored and based on theory guiding the behaviour change process to address and remove essential barriers. Another strength was that the study included physiotherapists and chiropractors from rural and urban areas with varying levels of experience and postgraduate education. This diversity of demographic variables in the sample ensured the generalizability of the results. However, the volunteer participation in the study may have included physiotherapists and chiropractors with a greater-than-average interest in a biopsychosocial treatment approach. Thus, the study population profile may explain why the implementation programme improved target behaviours, professional identity, and clinic culture.

A limitation of the study was the use of self-reported measurements for all outcomes. A study by Fritz et al. showed that self-reported clinical behaviours were overestimated compared to the observed behaviours [62], therefore, it is recommended to use observations rather than self-reported measurements [62]. However, observation methods require more resources; therefore, another option is to use patient journals. A review by Bérubé found changes in physiotherapists' beliefs, attitudes, and skills using self-reported measures; however, no change was found using patient journals [53]. Whether the positive self-reported measurements were caused by social desirability bias, with the physiotherapists reporting what they believed the researchers requested, or whether the physiotherapists' behaviour change was not adopted in the patient journals, i.e., the physiotherapists did not report their actual behaviour, is unknown. Hence, measuring behaviour is challenging, and all three methods (self-reported surveys, observations, and patient journals) cause uncertainties. Additionally, since no power calculation was performed, the study may be "underpowered", and no multiple comparisons were taken into account in the analysis, there is a risk of a Type 1 error. Finally, a limitation of the study is that no control group was included, e.g., the more common passive implementation strategies, such as after-work meetings for HCPs in which clinical guideline recommendations are presented.

## Conclusion

The tailored theory-driven implementation programme seems to enhance the adoption of key recommendations from the LBP guidelines among Danish physiotherapists and chiropractors working in primary care. The adoption was likely achieved because the programme positively affected participants' sense of being more skilled and more confident in adopting a biopsychosocial approach. Also, the programme positively affected the biopsychosocial culture at the clinics. Thus, the mechanisms linking contextual barriers with intervention functions and behaviour techniques that lead to the five strategies have been predominantly successful. Still, not all participants adopted the target behaviours, and future studies should further examine the mechanisms underlying non-adoption. A contributing factor to non-adoption could be the programme's feasibility, which participants assessed as only moderate. Still, most participants found the various strategies to be partly or overall acceptable and appropriate, indicating the need to adjust the programme structure rather than the content and format of specific strategies.

## Supplementary Information

Supplementary Material 1.

Supplementary Material 2.

Supplementary Material 3.

Supplementary Material 4.

Supplementary Material 5.

Supplementary Material 6.

## Data Availability

The datasets generated and/or analysed during the current study are not publicly available due the European GDPR, making data available impossible. Also, the data are in Danish and it is therefore impossible to make them comprehensibly accessible to the readers of Implementation Science Communications but are available from the corresponding author on reasonable request.

## Abbreviations

LBP: Low back pain
EBP: Evidence-based practice
HCPs: Healthcare professionals
COM-B: Capability, Opportunity, Motivation- Behaviour
