# Supplementary material for: Can a tailored implementation programme enhance the adoption of guideline-adherent behaviour in physiotherapists and chiropractors managing patients with low back pain? An implementation study
Source: Implement Sci Commun. 2025 Dec 6;7:7. doi: 10.1186/s43058-025-00820-y (PMC12797829; doi:10.1186/s43058-025-00820-y)
Supplement: Supplementary file 2 — Supplementary Material 2. [file 43058_2025_820_MOESM2_ESM.docx]

Additional file 2: Screening of patients' THOUGHTS

**Patients' thoughts can cover several elements:**

- The patient may have inappropriate and mistaken thoughts about the condition and be affected by anxiety and worry: fear of having a critical condition or fear of something in the back will break during movement.
- The patient may have catastrophizing and/or negative expectations of prognosis with concerns about not returning to everyday life.
- The patient may be affected by a state of mind of anxiety and worries leading to stress, negative/depressive thoughts and bad mood.

Key questions:

- **What do you think is the cause of your condition?**

Additional questions:

- When your pain worsens, what do you think is happening in your back? Does it worry you?
- What do you know about your back pain?
- What do you expect will help you?
- What are your thoughts about the future concerning your back pain?
- I hear that the pain affects your life in many ways - how much does it affect your mood?

- - - - - - - - - - - - - - - - - - - - - - - - - - - - - - - - - - - - - - - - - - - - - - - - - - - - - - - - - - - - - - - - - - - - - - - - - - - - - - - - - - -

Examples of several screening questions:

Thoughts on the condition:

**Often, when you are in pain, you have an idea of what is causing the pain. Even if you are not a doctor, chiropractor, or physiotherapist, do you have an idea of ​​what is wrong with your back?**

• What do you think is the cause of your condition?

• Has anyone explained what is causing your back pain?

- What do you think of that explanation?
- Does that explain your symptoms?
- Does it make sense to you?

• What do you know about your back pain/have you been given a diagnosis?

• When your pain worsens, what do you think is happening in your back? Does it worry you?

• What do you think worsens/improves your pain/condition?

Fear avoidance:

If you are doing something and you feel worsening/increased pain in your back, are you afraid that you may worsen your condition?

• Do you stop the activity or continue?

• What makes you avoid or change the activity?

- Do you think that pain is always a sign that you are worsening your condition and harming yourself?
- What advice have you received about activity and work?
- What advice have you been given about exercises?

Catastrophe thinking / Negative thoughts about prognosis

**Do you get very worried when your back pain worsens?**

• How do you think your back condition is in about 5 years from now - with pain and work/activity?

• How do you see your future / how do you think it will go?

• What are your thoughts about the future concerning your back pain? Do you believe you will recover?

• What are your thoughts on prognosis? Do you believe you will get better?

• Do you think a lot about your back pain?

Mood/state of mind:

**Does your pain ever make you in a bad mood/depressed?**

• When you have had pain for a long time, people often say that it affects your mood/state of mind. Have you experienced it?

• How do you feel about everything that has happened to your current situation?

• Is there something that makes you sad, or do you worry about the pain?

• How is your mood?

## Examples of Patient education THOUGHTS: description of the three boxes under YES

1) Examples of asking probing questions: What is the concern? With the deepening questions, we want to understand the concerns: What are they about? We need this knowledge to be able to target how we can best help the patient have more appropriate thoughts. Is it, for example, concerns/negative thoughts about the future, anxiety about being seriously ill, or a patient characterized by worries and bad mood due to stress in the environment?

*• Try to describe the last concrete episode where you experienced being worried...*

*• Take me back to… where you became concerned about… what did you experience?*

*• When you say you are worried about... what are you worried about will happen?*

*• When you avoid... what are you worried will happen?*

*• When you... (e.g. play golf/sit down) what are you worried about happening to your back? Or what do you imagine is happening in your back?*

*• When you say you are worried about the future, what/how do you imagine your future?*

*• You say you are worried about something seriously wrong with your back; what are you worried about? What have you been told about your back condition?*

**2) Examples of mirroring the patient's thoughts**

When you mirror the patient's thoughts, imagine holding a mirror up in front of the patient, mirroring the patient's thoughts so that the patient is made aware of the inappropriate thoughts.

*• Repeat/summarize the patient's thoughts: If I have to pick up, I hear that you are worried about... because... Is this understood correctly?*

*• If I understand correctly, you have stopped… because…*

*• How much has it helped you to avoid…?*

*• Do you want to be able to… again?*

### 3) Examples of offering a reassuring dialogue

When you offer reassuring dialogue, you can imagine that you are turning the mirror so that the patient sees his condition from a new angle. You thereby guide the patient to have a more appropriate way of thinking. This can be done by offering to share your knowledge about back pain. The patient is thereby put into a reflective process. It is important that the reassuring dialogue does not neglect the patient's experiences or is characterized by positive thinking. Patients can rarely find something positive in being in pain or being limited. Instead, it must try to de-dramatize the patient's thoughts about the condition and create understanding and reassurance.

*• Want to hear what I think about your condition?*

*• Can I share what I know about back pain with you?*

*• We know that our thoughts about our pain have a great influence on how we experience the pain. For example, we can all feel an itch on the scalp when someone mentions lice. This is also the case with our back pain. Therefore, your thoughts about your back affect your experience of the pain. When you feel pain in your back, it is rarely a sign that something in your back is broken. It is more often a sign that something is stiff and needs to be moved or that you are protecting your back and will move very stiffly.*

*• You can imagine… (give an example like in the video with the hand)*
