# Supplementary material for: Can a tailored implementation programme enhance the adoption of guideline-adherent behaviour in physiotherapists and chiropractors managing patients with low back pain? An implementation study
Source: Implement Sci Commun. 2025 Dec 6;7:7. doi: 10.1186/s43058-025-00820-y (PMC12797829; doi:10.1186/s43058-025-00820-y)
Supplement: Supplementary file 3 — Supplementary Material 3. [file 43058_2025_820_MOESM3_ESM.docx]

###### The patient's behaviour can involve several elements:

- The patient may have decreased/increased activity level (activities carried out individually) and participation level (activities carried out jointly with others), which may concern leisure and work.
- The patient may be characterised by decreased/increased sleep (quality and quantity).
- The patients may have lost their belief in managing the condition themselves.

During the screening, you examine the patient's behavioural strategies: Are they appropriate for their condition and progress, and do they believe in their ability to handle the condition?

Key questions:

- **How is your activity level - do you do more or less than usual?**

Additional questions:

- How is your activity level - is it as usual, or have you stopped doing some activities? (Why)
- Which activities are valuable for you in your life, which are painful, or do you altogether avoid them?
- Are you with your friends/family/social networks to the same extent as usual?
- How is your ability to work affected by your pain?
- Have you previously been on sick leave from work due to back pain?
- What do you do to manage the back pain?
- How do you sleep at night?

- - - - - - - - - - - - - - - - - - - - - - - - - - - - - - - - - - - - - - - - - - - - - - - - - - - - - - - - - - - - - - - - - - - - - - - - - - - - - - - - - -

Examples of several screening questions:

Activity and participation level (free time):

When you are in pain, you sometimes change your activity level and your social life. Is yours as usual, or do you do more or less?

- Why?

• What prevents you from doing/participating in … (activity/participation) as usual?

• Do you want to resume … (the activity/participation)?

• What activities and social events do you want to come back to?

Activity level (work):

**What functions do you have at work - are there any functions/tasks you no longer perform?**

- Why?
- Do you have other functions that you perform instead? Which ones?

• Do you want to return to your original work functions/tasks?

Sleep:

**How much sleep do you get on average each night?**

• How much sleep do you think is optimal for you?

• How is your sleep?

• Do you have trouble sleeping?

- Why?
- What do you do when you have trouble sleeping?

• Is it your experience that your sleep impacts your condition?

Behavioural strategies/ability to manage the condition:

**What are you currently doing to cope with your pain as best as possible?**

• How well do you feel you can manage the condition?

• If you are doing something and you feel your back getting worse, what do you do?

- What do you do when you get more pain?

• Is there anything you can do to help yourself?

- Is there anything you can do to ease or reduce your symptoms?

• Can you organise the day to do what is best for you?

Additional file 3: Screening of patients' behaviour

**Examples of Patient education: Behaviour: description of the three boxes under YES**

### 1. Examples of involving the patient in a new strategy

Examples of engaging questions:

By involving the patient in a new, more appropriate behavioural strategy, they will be more motivated for the upcoming change. The involvement also allows you to adapt the guidance to the patient's daily life and goals for the course. Now that you have had a reassuring dialogue, it is essential to ask the patient what they think could be an alternative behavioural strategy. It is also necessary to ask whether they are motivated or feel unsafe at having to resume or reduce their activity level*.*

*Decreased level of activity and participation:*

*• What are your thoughts about resuming... (leisure activity, work, participation)?*

*• Have you thought about how to get back to... that you care about...?*

*• How will you achieve your goal of resuming/retaining...?*

*• What is realistic for you regarding doing the exercises/training program: what time of day/week? How often?*

Increased level of activity and participation:

- What do you think about lowering your activity level?
- How much do you think will be a good level?
- What will be realistic for you?

**2. Examples of trying the movements that the patient is concerned about (in the consultation)**

(for patients with a reduced level of activity and participation)

By exposing the patient to positions and movements that they are worried about and possibly stopped, the patient is exposed to small stimuli that can accustom and calm the patient's thoughts and nervous system. At the same time as the patient is exposed, it is important that you ask about the patient's thoughts. Feel free to divide the activities into positions and smaller movements and use an exposure ladder. It can be helpful to get the patient to focus on breathing.

- Our back is a robust structure that needs movement. You have protected your back over a long period of time and therefore not moved it as much. Are you ready to try moving it more naturally again?
- We have talked about how movement and using your back is important and good for your back. How about trying to move it quietly again?
- One way to get to... (activity) again is to quietly turn your body to... is not dangerous and does not hurt your back. Are you willing to try… if we break it up into small movements?

**3. Examples of creating a concrete strategy for how to change the behaviour (between consultations)**

Based on the patient's goals and wishes, a concrete plan is made for how the behaviour is recovered or reduced in the real world - that is, which plan the patient sends home with. Patients with a decreased level of activity and participation may still need to be sent home with partial activities, depending on the individual patient's condition. Feel free to let the patient come up with suggestions on how to work with the exposure or reduction at home.

For example, ask the patient whose goal is to drive again:

You have said that you have not driven a car for several months because you have pain when sitting. Today, you tried sitting with your back bent, and your legs outstretched, expressing that it felt okay.

- What do you think about trying to drive again?
- Do you want the courage to try driving a car?
- How far do you think you are ready to drive?
- Is there a place you wish you could drive to? So, can you set it as a goal to be able to drive there before our next meeting? Start with a shorter trip first.

**Reassuring information and guidance:**

If you feel pain or worry when you go for a drive, you can think that sitting in a car is perfectly okay for your back. At the same time, try to take a few deep breaths. You can also adjust the seat or take a pillow in the back if it provides a more comfortable sensation.

**Other examples of concrete strategies:**

- If you get pain when you have to try to ... (activity), what do you think your pain might be an expression of? What can you do if you experience pain?
- What do you think about trying to carry a shopping bag to the car before your next visit? What could you imagine starting to lift in the bag? What do you think about having to lift something again?
- Can you do the same exercise at home as you did here today? How often will be realistic for you?
