# Supplementary material for: Can a tailored implementation programme enhance the adoption of guideline-adherent behaviour in physiotherapists and chiropractors managing patients with low back pain? An implementation study
Source: Implement Sci Commun. 2025 Dec 6;7:7. doi: 10.1186/s43058-025-00820-y (PMC12797829; doi:10.1186/s43058-025-00820-y)
Supplement: Supplementary file 4 — Supplementary Material 4. [file 43058_2025_820_MOESM4_ESM.docx]

**The patient's context can handle several elements:**

- *The patient may experience a lack of or inappropriate support from family, friends and/or the workplace (what physical demands and expectations the context places on the patient), social conditions (does the context support the patient in maintaining their social life) and attitudinal conditions (what are the context's thoughts about the patient's condition)*
- *The patient may be in a situation where external circumstances cause strain/stress, such as serious illness in the family, divorce, work stress, life crises, or children who are not thriving*

Key question

- **How do your family, friends and work react to your situation/condition?**

Additional questions:

- How do your surroundings react to your back pain?
- How do your employer and colleagues respond to your back pain?
- On sick leave: Do you think you will return to work? When? What does it take?
- Is your pain putting you in financial difficulty?
- Are there any insurance claims related to your condition?
- Are you having any meetings at the Job Centre? What is your experience of the meetings?
- Do you experience other forms of strain/stress in your life?

- - - - - - - - - - - - - - - - - - - - - - - - - - - - - - - - - - - - - - - - - - - - - - - - - - - - - - - - - - - - - - - - - - - - - - - - - - - - - - - - -

Examples of several screening questions:

Support from family and friends:

### Do you experience support from your family and friends to manage your pain?

- If yes, how? If not, what are you missing?

• Do you find that your family and friends make physical demands, such as daily chores or walks?

- Do you find it supportive?

• Do you have any experience of whether your family and friends try to motivate you to maintain your social life?

• What do your family and friends think about your condition?

Support from work:

**Do you experience a balance between your ability to work and what is required at your job?**

• Do you want to keep your job?

• How do you feel at work?

• How is your relationship with your manager/employer/colleagues?

• Do you experience support from your colleagues to manage your pain?

External circumstances:

• Can you change the load/stress in your life right now?

• Are you worried about your finances?

• Is your condition reported to the insurance company? Is there a case?

- If yes, is it something that fills you? How?

###### Additional file 4: Screening of patients' context

**Examples of** **Patient education: CONTEXT: description of the three boxes under YES**

1. **Examples of asking in-depth questions: What are the stress factors/the reactions from the surroundings?**

With the in-depth questions, we aim to identify any lack of/inappropriate support or external circumstances. We need this knowledge to target how we can best help the patient understand and interact with the environment. Is it, for example, concerns of those closest to you or a lack of knowledge on the part of the employer, or is the patient affected by unhappiness or illness in the family?

- *Try to describe how your experience... (spouse, friend, colleague, manager, others) reacts when you are in pain. What do they say and do?*
- *When you say that your… (spouse, friend, colleague, manager, or others) is worried, do you know what they are concerned about?*
- *When you say you are not ready to resume your work, what is it about?*
- *When you say you are worried about your finances, what are you worried will happen?*
- *Do you experience a connection between... (external load/stress, e.g. illness, divorce, workload) and your pain? How? (do you have more pain, sleep less, etc.)*

1. **Examples of involving the patient in a new strategy with the environment:**

By involving the patient in a dialogue about the contextual factors, they are put into a reflexive process where they begin to become aware of the factors that influence their condition. The involvement in the new strategy involves investigating whether the patient has been in dialogue with his surroundings about which support they perceive as helpful. For example, has the patient discussed with his colleagues how they can help them return to work? If it is about external circumstances that cannot be changed, the involvement will instead focus on helping the patient reflect on the significance of these circumstances.

- *Have you expressed to... (spouse, friend, colleague, manager, others) how you want their support?*
- *Have you expressed how they can best help you to those around you?*
- *Do you think changing your... (spouse, friend, colleague, manager, others) reaction and support is possible? If so, how?*
- *Have you expressed to… (spouse, friend, colleague, manager, others) how it makes you feel when they express concern?*
- *Do you talk to… (spouse, friend, colleague, manager, others) about your condition?*
- *What are your thoughts on talking to your… (spouse, friend, colleague, manager, others) about their support?*
- *Do you think you can change the situation with... (divorce, illness, workload, other)?*
- *What effect do you think... (divorce, illness, workload, other) have on your condition?*
- *Do you need help from me or others?*

1. **Examples of creating a concrete strategy and identifying opportunities for support:**

Based on the patient's experiences and wishes, a concrete strategy is developed to change the support from his surroundings. Feel free to let the patient come up with suggestions on how to enter into a dialogue with their surroundings. If the patient thinks he cannot change the contextual factors, opportunities for support are identified instead. As a clinician, you can help the patient by offering a family member reassurance through a dialogue or investigating options for support from a retention consultant. Suppose the patient is affected by external circumstances that cannot be changed. In that case, the strategy can instead focus on helping the patient accept the circumstances and explore their options.

- *What do you think about conversing with... (spouse, friend, colleague, manager) about how they best support you in... activity or managing your pain?*
- *Make a plan together with the patient to enter into a dialogue with his surroundings: At (insert time), I will (insert communication) with (insert person)*
