# Supplementary material for: Can a tailored implementation programme enhance the adoption of guideline-adherent behaviour in physiotherapists and chiropractors managing patients with low back pain? An implementation study
Source: Implement Sci Commun. 2025 Dec 6;7:7. doi: 10.1186/s43058-025-00820-y (PMC12797829; doi:10.1186/s43058-025-00820-y)
Supplement: Supplementary file 5 — Supplementary Material 5. [file 43058_2025_820_MOESM5_ESM.docx]

###### Additional file 5: Example of data collection of acceptability, appropriateness, and feasibility sent by weekly text messages

| **Text message week 1** | **Answer options** | **Outcomes**  *Not visible for the participants* |
| --- | --- | --- |
| Did you participate in the TAK course's first webinar that was just held?  If no, they were asked:  Have you subsequently watched the webinar? | - Yes - No | Feasibility |
| Were you satisfied with the content of the webinar? | - Yes, overall satisfied - Yes, partially satisfied - No, not satisfied - Do not know | Acceptability |
| Was the webinar an appropriate way to gain knowledge about why psychological and social issues are important to include in your assessment and treatment? | - Yes, overall appropriate - Yes, partly appropriate - No, not appropriate - Do not know | Appropriateness |
| **Text message week 2** | | |
| Have you seen the e-learning video about "Expectation alignment"? | - Yes - No | Feasibility |
| Were you satisfied with the content of the e-learning video? | - Yes, overall satisfied - Yes, partially satisfied - No, not satisfied - Do not know | Acceptability |
| Have you completed the communication exercise on "expectation alignment" with one or more of your colleagues? | - Yes - No | Feasibility |
| Was the communication exercise an appropriate way to practice expectation alignment? | - Yes, overall appropriate - Yes, partly appropriate - No, not appropriate - Do not know | Appropriateness |
| **Text message week 10** | | |
| Have you participated in a peer learning session focusing on Patients’ Behaviour? | - Yes - No | Feasibility |
| Were you satisfied with the procedure and the note sheet used to guide the peer-learning session? | - Yes, overall appropriate - Yes, partly appropriate - No, not appropriate - Do not know | Acceptability |
| Do you think peer learning was an appropriate way to get feedback on your communication with the patient? | - Yes, overall appropriate - Yes, partly appropriate - No, not appropriate - Do not know | Appropriateness |
