# Supplementary material for: Can a tailored implementation programme enhance the adoption of guideline-adherent behaviour in physiotherapists and chiropractors managing patients with low back pain? An implementation study
Source: Implement Sci Commun. 2025 Dec 6;7:7. doi: 10.1186/s43058-025-00820-y (PMC12797829; doi:10.1186/s43058-025-00820-y)
Supplement: Supplementary file 6 — Supplementary Material 6. [file 43058_2025_820_MOESM6_ESM.docx]

**Additional file 6: Baseline and 16 weeks follow-up data**

**Target Behaviours**

Do you screen your patients' psychological and social issues?

|  |  |  | **Follow-up** | |  |
| --- | --- | --- | --- | --- | --- |
|  |  | Not at all | Low | Moderate | High |
|  | Not at all | 0 | 0 | 0 | 0 |
| **Baseline** | Low | 0 | 1 | 11 | 2 |
|  | Moderate | 0 | 1 | 37 | 13 |
|  | High | 0 | 0 | 3 | 9 |

Do you guide your patients in managing their psychological and social issues?

|  |  |  | **Follow-up** | |  |
| --- | --- | --- | --- | --- | --- |
|  |  | Not at all | Low | Moderate | High |
|  | Not at all | 0 | 0 | 0 | 0 |
| **Baseline** | Low | 0 | 2 | 8 | 0 |
|  | Moderate | 0 | 3 | 29 | 11 |
|  | High | 0 | 0 | 5 | 4 |

**Biopsychosocial professional identity**

Do you believe it is your *role* to involve your patients' psychological and social issues?

|  |  |  | **Follow-up** | |  |
| --- | --- | --- | --- | --- | --- |
|  |  | Not at all | Low | Moderate | High |
|  | Not at all | 0 | 0 | 0 | 0 |
| **Baseline** | Low | 0 | 0 | 1 | 2 |
|  | Moderate | 0 | 0 | 19 | 17 |
|  | High | 0 | 0 | 5 | 33 |

Do you have the sufficient *skills* to screen your patients' psychological and social issues?

|  |  |  | **Follow-up** | |  |
| --- | --- | --- | --- | --- | --- |
|  |  | Not at all | Low | Moderate | High |
|  | Not at all | 0 | 0 | 0 | 0 |
| **Baseline** | Low | 0 | 5 | 27 | 4 |
|  | Moderate | 0 | 3 | 24 | 10 |
|  | High | 0 | 0 | 2 | 2 |

Do you have the sufficient *skills* to guide your patients in managing psychological and social issues?

|  |  |  | **Follow-up** | |  |
| --- | --- | --- | --- | --- | --- |
|  |  | Not at all | Low | Moderate | High |
|  | Not at all | 0 | 0 | 0 | 0 |
| **Baseline** | Low | 0 | 1 | 11 | 0 |
|  | Moderate | 0 | 0 | 16 | 6 |
|  | High | 0 | 0 | 2 | 2 |

Do you *feel confident* in involving your patients' psychological and social issues?

|  |  |  | **Follow-up** | |  |
| --- | --- | --- | --- | --- | --- |
|  |  | Not at all | Low | Moderate | High |
|  | Not at all | 0 | 0 | 0 | 0 |
| **Baseline** | Low | 0 | 1 | 12 | 1 |
|  | Moderate | 0 | 3 | 27 | 22 |
|  | High | 0 | 0 | 1 | 9 |

Are you *interested* in involving your patients' psychological and social issues?

|  |  |  | **Follow-up** | |  |
| --- | --- | --- | --- | --- | --- |
|  |  | Not at all | Low | Moderate | High |
|  | Not at all | 0 | 0 | 0 | 0 |
| **Baseline** | Low | 0 | 1 | 5 | 0 |
|  | Moderate | 0 | 1 | 12 | 10 |
|  | High | 0 | 0 | 9 | 37 |

**Biopsychosocial culture**

Do you experience your colleagues involving their patients' psychological and social issues?

|  |  |  | **Follow-up** | |  |
| --- | --- | --- | --- | --- | --- |
|  |  | Not at all | Low | Moderate | High |
|  | Not at all | 0 | 0 | 0 | 0 |
| **Baseline** | Low | 0 | 7 | 8 | 1 |
|  | Moderate | 0 | 2 | 25 | 8 |
|  | Høj | 0 | 0 | 1 | 7 |

Do you engage in or request *professional back-and-forth* with your colleagues about your patients’ psychological and social issues?

|  |  | **Follow-up** | | |  |
| --- | --- | --- | --- | --- | --- |
|  |  | Not at all | Low | Moderate | High |
| **Baseline** | Not at all | 0 | 3 | 0 | 0 |
|  | Low | 1 | 19 | 12 | 3 |
|  | Moderate | 1 | 3 | 22 | 8 |
|  | High | 0 | 0 | 0 | 5 |
